# Direct Z-scheme of n-type CuS/p-type ZnS@electrospun PVP nanofiber for the highly efficient catalytic reduction of 4-nitrophenol and mixed dyes†

**DOI:** 10.1039/d2ra01476a

**Published:** 2022-05-31

**Authors:** Elvri Melliaty Sitinjak, Indra Masmur, New Vita Mey Destty Marbun, Poltak Evencus Hutajulu, Golfrid Gultom, Yenny Sitanggang

**Affiliations:** Department of Chemical Engineering, Politeknik Teknologi Kimia Industri Medan-20228 Indonesia; Department of Chemistry, Faculty of Mathematics and Natural Sciences, Universitas Sumatera Utara Medan-20155 Indonesia intar76@yahoo.com; Department of Palm Oil Agribusiness, Politeknik Teknologi Kimia Industri Medan-20228 Indonesia; Department of Mechanical Engineering, Politeknik Teknologi Kimia Industri Medan-20228 Indonesia

## Abstract

Environmental pollution has been the most critical issue on earth due to many factors, particularly the industrial chemical waste, which can be detoxified by photocatalytic methods. In this study, we demonstrate the fabrication of an electrospun composite nanofiber embedded with n-type CuS and p-type ZnS in partially carbonized-PVP nanofibers, so-called Z-type-CuS/ZnS@PVP nanofibers, to reduce 4-nitrophenol to 4-aminophenol and degrade the mixed dyes of methylene blue, rhodamine B, and methyl orange. The Z-type-CuS/ZnS@PVP nanofibers were prepared by an electrospinning method, followed by annealing at 180 °C and 400 °C under N_2_ atmosphere. As-prepared CuS/ZnS@PVP nanofibrous mats were characterized by SEM, XRD, PL, DRS, TPC, and EIS analyses. The results revealed that Z-type CuS/ZnS@PVP nanofibers have enhanced optical and electrochemical properties as compared with the CuS@PVP and ZnS@PVP nanofibers. Likewise, the Z-scheme was more beneficial for promoting the electron transfer as well as for delaying the photocarrier recombination. For the applications of CuS/ZnS@PVP nanofibers, the reduction of 4-nitrophenol to 4-aminophenol occurred within 2 h and the mixed-dye degradation occurred in 90 min in 5% MeOH aqueous solution under solar light irradiation. The CuS/ZnS@PVP nanofibers also possessed excellent stability, with more than 95% remaining after five recycle runs. The photocatalytic mechanism reaction is proposed, in which the mechanism was initiated by the adsorption of organic pollutants on the nanofiber matrix, followed by the photoreaction due to e^−^ and h^+^ in CuS/ZnS after light irradiation as well as from the generated radical species. Lastly, the inorganic photocatalyst embedded in the nanofiber matrix offered an easy recovery process with excellent degradation performance as well.

## Abstract 2

Z-type CuS/ZnS@PVP nanofibers are synthesized for the photocatalytic reduction of 4-nitrophenol and for mixed-dyes degradation under mild conditions.

## Graphical abstract

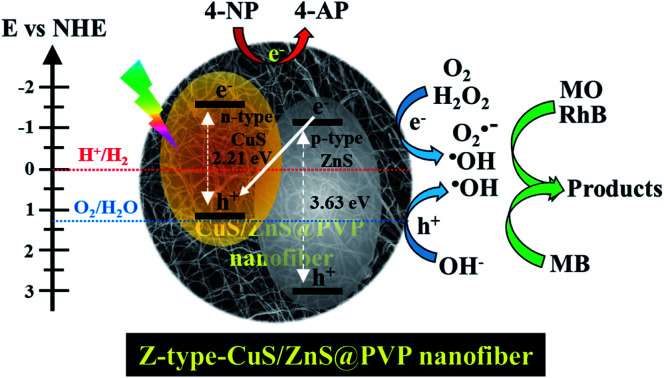

## 1. Introduction

In recent decades, the further development of industrialization has been growing rapidly and accordingly, the demand for chemicals in industries has also been increasing. Regardless of this, the discharge of untreated wastewater from industries into water bodies lead to a serious impact on our environment. The contamination in freshwater is mostly due to chemical pollutants in water, such as nitroaromatic compounds,^1^ which are widely used in many industrial applications (*e.g.*, fungicides, insecticides, and dyestuffs). Unfortunately, nitroaromatic compounds are well known for their high toxicity and stability in water even at low concentration.^2–5^ The allowed concentrations of 4-nitrophenol in the environment range from 1 ppb to 20 ppb, as determined by the Environmental Protection Agency (EPA).^2^ Accordingly, several techniques, such as Fenton oxidation, photocatalysis, and adsorption, have been reported to detoxify contaminated water for ensuring a better environment.^6–8^ The reduction of 4-nitrophenol has also been achieved using conventional chemical methods with Fe–HCl and Sn–HCl; however, the processes yield a lot of waste byproducts (metal oxide), which are not beneficial either economically or environmentally.^9^ On the other hand, the textile, food, and paper industries widely use organic dyes. These industries possibly contribute a huge amount of dye wastes to the water bodies in our environment. Generally speaking, dye waste is usually carcinogenic, non-degradable, and harmful, even at a low concentration.^10^ Further efforts toward removing dye wastes are urgently needed to keep our environment clean and healthy.

Moreover, nanocatalysts with a high surface area and good stability have attracted a lot of attention for the efficient conversion of 4-nitrophenol to 4-aminophenol under mild conditions.^11^ In these past few years, several materials have been developed for the reduction of 4-nitrophenol to 4-aminophenol, including through the use of carbon-based materials, metal oxides, and metal nanoparticles.^12–14^ Among all the reported materials, the semiconductor materials are believed to offer a powerful catalytic route. For example, the three-dimensional (3D) ZnO/Ag nanocomposites have been reported for 4-nitrophenol reduction using NaBH_4_.^15^ In another study, Luo *et al.* fabricated the Fe@Fe_2_O_3_/HA core–shell system for nitrophenol reduction. They also found that the redox cycle of Fe^3+^/Fe^2+^ accelerated the generation of OH˙ to progress the Fenton reaction.^16^ However, studies using metal sulfide materials have been seldom explored for nitrophenol reduction. On the other hand, the use of a reducing agent, such as NaBH_4_, including its byproduct of NaBO_2_ could lead to environmental problems, which would cause other chemical pollution that could cause damage to human health.

In the photocatalysis field, there are still several challenges remaining, such as the fast recombination between the photogenerated electrons and holes, limited active surface area, ease of catalyst collection, and its reusability.^17–19^ Accordingly, the utilization of substrates like electrospun nanofibers or metal foam is expected to solve the aforementioned challenges. The high porosity networks, extremely high aspect ratio, and good mechanical properties of nanofibers make them suitable as a support to grow photocatalysts.^20^ For the photocatalysts, a Z-scheme concept was designed with n-type CuS and p-type ZnS. The reasons for choosing CuS and ZnS are because these semiconductors exhibit a suitable bandgap position, which means they are able to absorb in both the visible-light and UV region wavelengths due to the band gap energy of CuS (∼2.2 eV) and ZnS (∼3.6 eV). On the other hand, they are also well known for their catalytic activities, abundances, and economical friendliness.^21,22^

To the best of our knowledges, no studies have yet reported the n-type CuS and p-type ZnS on partially carbonized-PVP nanofibers for photocatalytic 4-nitrophenol reduction and mixed-dyes degradation. Therefore, in this study, we report a facile preparation of Z-type-CuS/ZnS@PVP nanofibers prepared by electrospinning and annealing methods for photocatalytic applications. The Z-scheme in the CuS/ZnS@PVP nanofiber offers a synergetic effect that can efficiently suppress the recombination of e^−^ and h^+^; therefore improving the photocatalytic activities either for 4-nitrophenol reduction or mixed-dyes degradation. The photocatalytic activities toward the reduction of 4-NP to 4-AP under solar light irradiation was investigated in 5% MeOH solution and the proposed mechanism was discussed as well. The reason for the immobilization of CuS/ZnS in the PVP nanofibers was not just to prevent the common leaching issue of photocatalysts, but also for the ease of reusability.

## 2. Materials and methods

### 2.1. Chemicals

PVP (average molecular weight = 40 000 g mol^−1^), 4-NP (purity 99%), copper(ii) acetate hydrate ((CH_3_COO)_2_Cu·H_2_O, purity 99.5%), zinc(ii) acetate ((CH_3_COO)_2_Zn, purity 97%), and thioacetamide (C_2_H_5_NS, purity 99%) were purchased from Sigma-Aldrich and used as received without further purification. Common solvents, such as ethanol and methanol, used for the preparation of the electrospinning doped and photocatalytic solution were obtained from commercial sources with the highest available purity. All the aqueous solutions were prepared with ultrapure water (resistivity 18.2 M cm^−1^) obtained from a Barnstead Nanopure water purification system.

### 2.2. Preparation of CuS/ZnS@PVP electrospun nanofibers

In a typical experiment, the acetate precursors of Cu (0.15 M) and Zn (0.15 M) and thioacetamide with a certain amount were dissolved in 5 mL aqueous solution containing 50% ethanol. After completely dissolving, 4 g of PVP powder was slowly added under stirring for 6 h. The resulting blue viscous solution was transferred to a 5 mL plastic syringe equipped with a stainless steel blunt-ended needle (21 gauge, *Ø* = 0.154 mm) as the spinneret. Electrospinning was carried out for 4 h under controlled relative humidity (40–50%) and temperature (35 ± 3 °C) at a 25 kV applied voltage and a 10 cm distance between the grounded drum collector and the spinneret. The polymer solution was continuously fed through a spinneret with a syringe pump at a constant flow rate of 0.1 mL h^−1^. Aluminum foil wrapped on the drum collector was used as a substrate for collecting the electrospun nanofibers. The deposited white fiber sheet was then carefully peeled off from the aluminum foil using a plastic tweezer and stored in an oven at 70 °C to minimize moisture absorption from ambient air. Thermal crosslinking *via* pre-oxidation was carried out in two-stage annealing at 180 °C and 400 °C for a total time of 4 h in an electric muffle furnace under a N_2_ atmosphere to obtain CuS/ZnS PVP nanofibers. For comparison purposes, CuS@PVP and ZnS@PVP nanofibers were also fabricated.

### 2.3. Material characterizations

The morphology and elemental composition of the fabricated CuS/ZnS@PVP nanofibers were examined by a field-emission scanning electron microscopy system (FESEM, JEOL JSM 7900F) and an Oxford Instruments INCA energy dispersive X-ray (EDX) detector with an accelerating voltage of 15 kV. Fiber diameter measurements were performed on SEM images using the ImageJ 1.48 software with at least 20 randomly chosen fibers. X-Ray diffraction (XRD) measurements were carried out on a Bruker D2 Phaser X-ray diffractometer using the Kα line of a Cu source (*λ* = 0.15418 nm) operated at 30 kV and 10 mA. Diffraction patterns were collected in the 2*θ* range of 20–80° with a step size of 0.05° and a counting time of 1 s per step. The optical and electrochemical properties (*e.g.*, photoluminescence, transient photocurrent, electrochemical impedance spectroscopy) of the prepared samples were analyzed using an Autolab potentiostat in a three-electrode system (working electrode: sample, counter electrode: Pt foil, and reference electrode: Ag/AgCl) in KCl 0.1 M solution.

### 2.4. Photocatalytic experiments

The photocatalytic reduction using Z-type-CuS/ZnS@PVP nanofibers was carried out to convert 4-nitrophenol to 4-aminophenol using solar light in 5% MeOH solution as a hole scavenger. In a typical experiment, 20 mg of CuS/ZnS@PVP nanofibers was put into a beaker glass containing 30 ppm 4-nitrophenol with a total volume of 250 mL. Prior to light irradiation (Newport, 300 W), the mixtures were magnetically stirred in the dark for 30 min to ensure the adsorption–desorption equilibrium between 4-nitrophenol and the surface of the catalyst. The reaction progress was monitored by measuring the absorbance spectra of aliquots of the reaction mixture taken at certain time intervals. The maximum wavelengths (*λ*_max_) corresponding to 4-nitrophenol and the reduced product, 4-aminophenol, were observed at 400 and 300 nm, respectively.

In the second photocatalytic experiment, Z-type-CuS/ZnS@PVP nanofibers were used to demonstrate the photocatalytic oxidation reaction, particularly for mixed-dyes degradation. The mixed dyes consisted of methylene blue, rhodamine B, and methyl orange, each with a concentration of 10 ppm. The photocatalytic experiments were conducted in a similar procedure with the photoreduction of 4-nitrophenol and were conducted in triplicate. The reusability of the nanofibrous catalysts was tested for five consecutive cycles of the photocatalytic reduction of 4-nitrophenol and mixed dyes under the same reaction conditions. The spent nanofibrous catalyst was recovered, washed thoroughly with water, and dried under vacuum before performing the next catalytic run.

## 3. Results and discussion

### 3.1. Surface morphology and phase identification

**Fig. 1:**
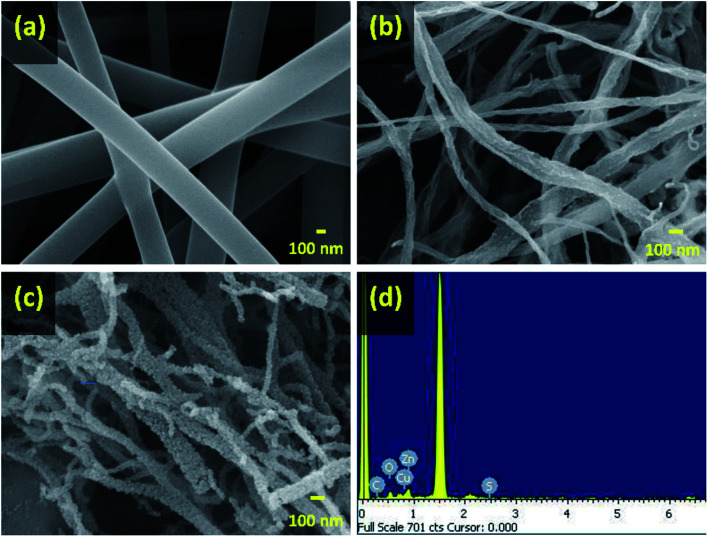
SEM images of (a) CuS/ZnS@PVP nanofibers, (b) CuS/ZnS@PVP nanofibers annealed at 180 °C, (c) CuS/ZnS@PVP nanofibers annealed at 400 °C under a N_2_ atmosphere with (d) its elemental composition by EDS analysis.

The surface morphology of the as-spun CuS/ZnS@PVP nanofiber is displayed in Fig. 1. The addition of metal salt into the polymer solution was also believed to affect the spinnability of the polymer jet since it improved the conductivity. The average diameter of the CuS/ZnS@PVP nanofibers decreased from 307 ± 12 nm to 129 ± 26 nm after annealing at 180 °C (Fig. 1b) and 400 °C (Fig. 1c). This could be attributed to the thermal decomposition of the polymer matrix that occurred during the annealing process, which also partially carbonized the nanofibers.^23,24^ For the thermal decomposition, the partial carbonization of CuS/ZnS@PVP nanofibers was confirmed by TGA analysis under a N_2_ atmosphere. Briefly, the weight percentages of both PVP and CuS/ZnS@PVP nanofibers retained ∼60% when calcined at 400 °C under a N_2_ atmosphere, as shown in Fig. S1 (ESI†). At the same time, Fig. 1c reveals that the surface of the CuS/ZnS@PVP nanofibers became rougher and agglomeration of the CuS and ZnS nanostructures was clearly visible after the annealing treatment, with the agglomerates also uniformly distributed along the nanofiber matrix. This result suggests that thermal annealing at 400 °C can induce the formation of crystalline metal sulfide phases (*i.e.*, CuS and ZnS), both of which are beneficial for catalytic reactions. Furthermore, the elements contained in the CuS/ZnS@PVP nanofiber by EDS analysis were found to be C, Cu, Zn, and S, with the atomic percentages of 47.4%, 11.7%, 13.1%, and 27.8%, respectively (Fig. 1d).

**Fig. 2:**
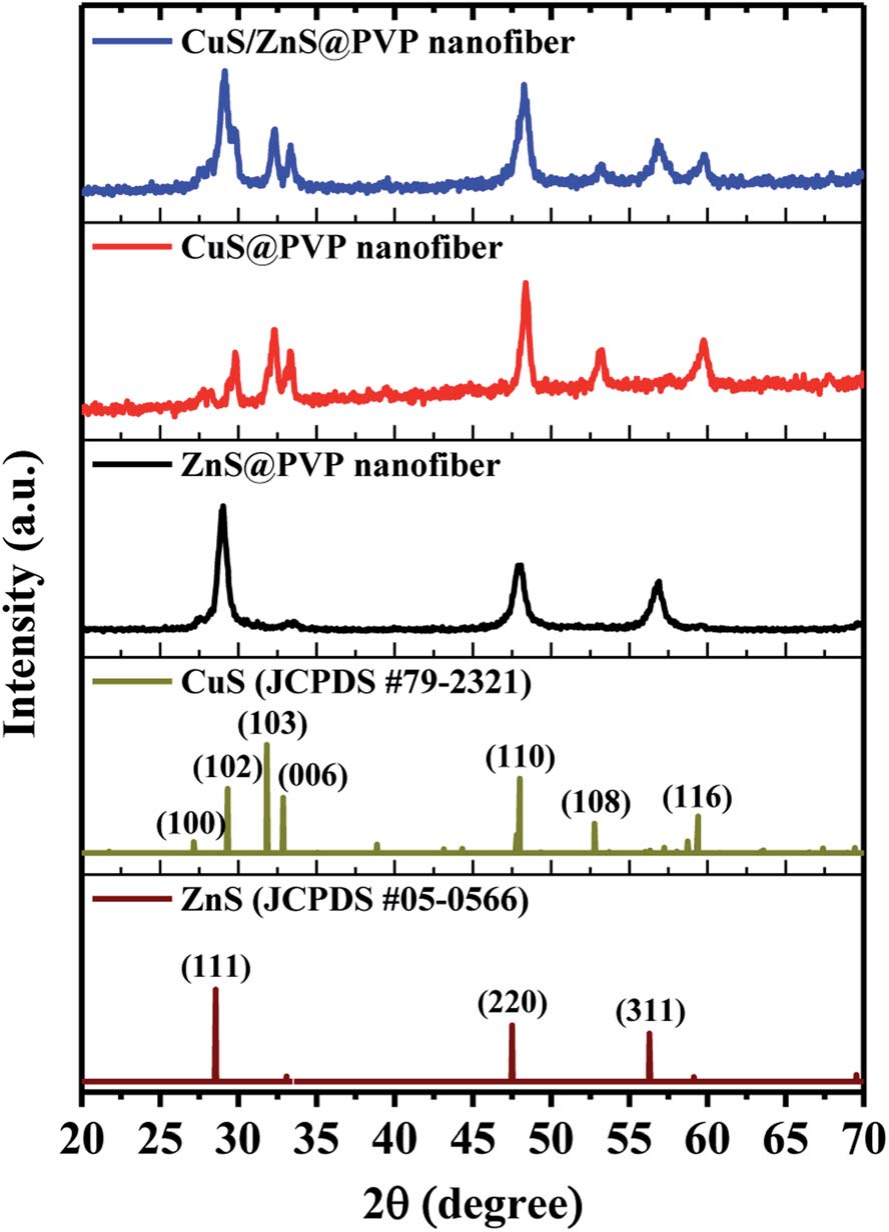
Diffractograms of CuS@PVP, ZnS@PVP, and CuS/ZnS@PVP nanofibers with the JCPDS standard cards of CuS (JCPDS no. 79-2321) and ZnS (JCPDS no. 05-0566).

The XRD patterns of the CuS@PVP, ZnS@PVP, and CuS/ZnS@PVP nanofibers are presented in Fig. 2. As can be seen in the figure, the CuS@PVP and ZnS@PVP nanofibers matched with the standard data of CuS (JCPDS no. 79-2321) and ZnS (JCPDS no. 05-0566). Meanwhile, the CuS/ZnS@PVP nanofiber sample contained both CuS and ZnS simultaneously (Fig. 2). On the other hand, there were no characteristic Bragg reflections associated with the crystal structures of metal oxides in the nanofibers, proving that the N_2_ purging during the annealing processes prevented the O atoms from entering the lattices of CuS or ZnS.

### 3.2. Optical and electrochemical properties

**Fig. 3:**
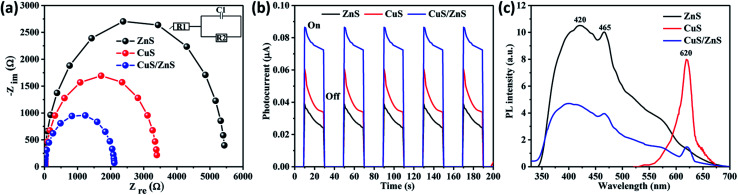
(a) Nyquist plots, (b) TPC response, and (c) PL spectra of CuS, ZnS, and CuS/ZnS incorporated in PVP nanofibers. All the measurements were carried out in KCl 0.1 M.

Electrochemical impedance spectroscopy (EIS) analysis was performed to study the resistance at the interfaces between the sample and electrolyte based on the charge transfer. Fig. 3a shows the semicircle Nyquist plot of CuS, ZnS, and CuS/ZnS decorated on PVP nanofiber fitted with equivalent Randles circuit (inset in Fig. 3a). The measured resistances of CuS@PVP and ZnS@PVP nanofibers were 3407 and 5485 Ω, respectively. After forming Z-type CuS/ZnS@PVP nanofibers, the resistance was significantly degraded to 2163 Ω, indicating an enhanced charge transfer from the catalyst to the electrolyte.^25^ As EIS analysis only showed the charge transfer by the resistance of the catalyst and electrolyte interface, the photocarriers (e^−^ and h^+^) generated under light irradiation also needed to be evaluated through the transient photocurrent (TPC) response technique. In this measurement, the light was switched on and off for 20 s repetitively for 5 cycles using the constant voltage mode. As can be seen in Fig. 3b, the photocurrent of the ZnS@PVP nanofibers was 0.04 μA, while it was 0.06 μA for the CuS@PVP nanofibers. The photocurrent of the CuS/ZnS@PVP nanofibers was dramatically enhanced to 0.087 μA after contacting with CuS/ZnS in the nanofiber. Upon light irradiation, the initial photocurrent spiked due to the abundant e^−^–h^+^ pairs and subsequently plunged to the steady condition after such a spike, indicating the recombination between e^−^ and h^+^ in the catalyst.^26^ In the TPC results, the spike shape of the CuS/ZnS@PVP nanofibers indicated a delay in e^−^–h^+^ recombination compared to the case with the CuS@PVP nanofibers and ZnS@PVP nanofibers (Fig. 3b). Moreover, the photoemission of the samples was determined by photoluminescence analysis to further study the recombination rate of e^−^ and h^+^. There were two photoemission peaks of ZnS at the wavelengths of 420 and 465 nm; meanwhile, the emission peak for CuS was located at 620 nm. Briefly, the lowest intensity exhibited by CuS/ZnS@PVP nanofibers revealed the slower recombination rate of photocarriers. All the results from EIS, TPC, and PL techniques were in a good agreement, indicating the composite of CuS/ZnS could synergistically improve the photocatalytic properties.

**Fig. 4:**
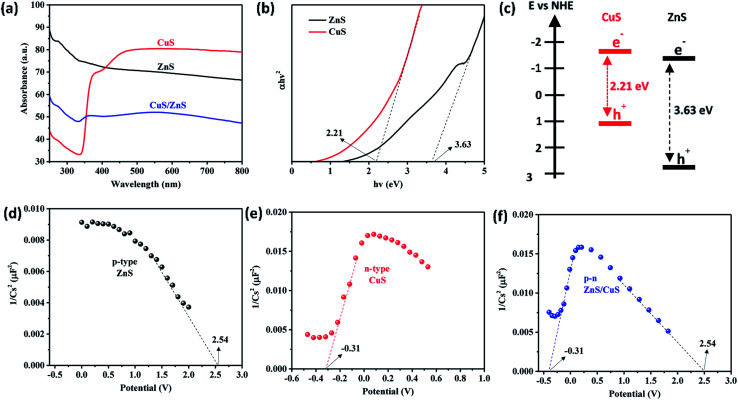
(a) Diffuse reflectance spectra, (b) Tauc plot, and (c) band diagram for the samples. Mott–Schottky plots of (d) p-type ZnS, (e) n-type CuS, and (f) CuS/ZnS@PVP nanofibers.

The diffuse reflectance spectra with their calculated Tauc plots are shown in Fig. 4a and b, respectively. It can be clearly seen that ZnS possessed wavelength absorption in the UV light region and CuS absorbed light in the visible-light region. The Tauc plots were calculated in order to determine the band gaps of the samples based on eqn (1). The band gaps of ZnS and CuS were calculated to be 3.63 and 2.21 eV, respectively. As expected, the CuS/ZnS@PVP composite nanofiber could absorb both UV and visible light from the solar light irradiation.

1 (*αhν*)^2^ = *k*(*E*_g_ − *hν*)

Next, Mott–Schottky measurements were conducted to confirm the type of semiconductors with an initial frequency of 200 MHz to a final frequency of 1 kHz. Fig. 4d–f show the characteristics p- and n-type profiles for ZnS, CuS, and CuS/ZnS@PVP nanofibers with the flat band potentials of +2.54 and −0.31 V, respectively. The Mott–Schottky measurements proved that the fabricated CuS/ZnS@PVP nanofibers were composed of a p–n heterojunction, with their band diagram drawn in Fig. 4c.

### 3.3. Photocatalytic activities

**Fig. 5:**
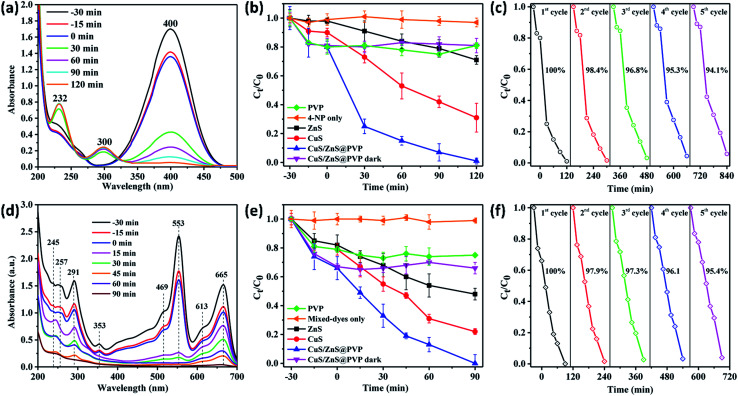
UV-vis spectra showing the conversion of (a) 4-nitrophenol and (d) mixed dyes. Plots of *C*_*t*_/*C*_0_*vs.* reaction time comparing PVP only, organic pollutants only, CuS, ZnS, and CuS/ZnS@PVP nanofibers to convert (b) 4-nitrophenol and (e) mixed dyes. The five-run reusability performances of CuS/ZnS@PVP nanofibers on (c) 4-nitrophenol, and (f) mixed dyes.

The investigation of the photocatalytic activity of CuS/ZnS@PVP nanofibers was performed to reduce 4-nitrophenol to 4-aminophenol in the presence of 5% MeOH as a hole scavenger under light irradiation. In a typical experiment, the CuS/ZnS@PVP nanofibers were immersed in 4-nitrophenol solution (30 ppm) for 30 min to let the adsorption–desorption progress. Once the equilibrium was achieved, the solution containing 4-nitrophenol and photocatalyst was irradiated with solar light. The result was monitored by UV-vis spectroscopy for showing the progress of 4-nitrophenol conversion to 4-aminophenol by the CuS/ZnS@PVP nanofibers, as shown in Fig. 5a. Initially, 30 ppm of 4-nitrophenol possessed an intensity of 1.7 (−30 min) with the peak located at 400 nm. The intensity of the peak gradually decreased due to surface adsorption of the CuS/ZnS@PVP nanofibers in the dark condition. Such a peak significantly degraded to an intensity of 0.4 at 30 min and, at the same time, new peaks appeared at 232 and 300 nm referring to 4-aminophenol. Finally, the 4-nitrophenol peak was completely reduced to 4-aminophenol with solar light irradiation for 2 h (Fig. 5a), with Fig. 5b showing the *C*_*t*_/*C*_0_*vs.* time plots for the different samples. It was found that there was only an adsorption–desorption process when using the catalyst-free PVP nanofibers. In the absence of a catalyst, the photostability with direct light irradiation toward 4-nitrophenol was insignificant (Fig. 5b). The *C*_*t*_/*C*_0_ of 4-nitrophenol conversion to 4-aminophenol was 0.72, 0.36, and almost zero, respectively, for ZnS, CuS, and CuS/ZnS@PVP nanofibers after light irradiation for 2 h. Meanwhile, there was no catalytic ability of CuS/ZnS@PVP nanofibers in the dark condition, as shown in Fig. 5b.

The conversion of 4-nitrophenol to 4-aminophenol was quantitatively confirmed by GC-MS analysis for the solution before and after the photocatalytic reaction, as shown in Fig. S2 (ESI†). The *m*/*z* peak = 139 in the solution before the photocatalytic reaction corresponded to the molar mass of 4-nitrophenol. Such a peak disappeared after the photocatalytic reduction after 2 h by CuS/ZnS@PVP nanofibers; instead, a new peak with *m*/*z* = 109 appeared, corresponding to 4-aminophenol as the photocatalytic product.^12^ Meanwhile, there was no reaction in the dark condition; instead, only adsorption–desorption processes took place. Reusability tests were also carried out to study the stability of the photocatalyst and the result is shown in Fig. 5c. The reusability percentage slightly decreased from 100% to 94.1% after five test runs, indicating that the inorganic catalyst in the nanofiber was stably embedded and not peeled off during the photocatalytic experiments.

In the second photocatalytic oxidation experiment, the p–n heterojunction CuS/ZnS@PVP nanofiber was used to degrade the mixed dyes (methylene blue, rhodamine B, and methyl orange). For information, methylene blue and rhodamine B are categorized as cationic dyes, while methyl orange is an anionic dye.^27,28^ The mixed dyes were allowed to absorb for 30 min on the p–n heterojunction CuS/ZnS@PVP nanofiber. The peaks located at 245, 291, 613, and 665 nm were related to methylene blue, while the peaks at 257, 353, and 553 nm corresponded to rhodamine blue, and the methyl orange peak appeared at 469 nm, as can be seen in Fig. 5c. Similar to the case of 4-nitrophenol, the catalyst-free PVP nanofiber only showed the adsorption–desorption process without any catalytic activities. The direct photolysis of the mixed dyes in the absence of the catalyst also revealed an unchanged concentration in the mixed-dyes concentration. The degradation of mixed dyes by the photocatalyst was first started by decolorizing the rhodamine B, followed by methyl orange and methylene blue. The *C*_*t*_/*C*_0_*vs.* time plot (Fig. 5d) showed a gradual degradation of the 30 ppm-mixed dyes by the Z-type CuS/ZnS@PVP nanofibers as compared to those of the CuS@PVP and ZnS@PVP nanofibers. The degradation of each dye is summarized in Table S1 (ESI†). In order to study the charge of particle surface, zeta potential measurements were carried out. As shown in Fig. S3 (ESI†), the results indicated that the zeta potential of pristine CuS/ZnS@PVP nanofibers was found to be negative (−21.47 mV). After mixing the photocatalyst with mixed dyes, the zeta potential shifted to −3.47 mV. This was because of the coulombic interaction of the cationic dyes (methylene blue and rhodamine B) in the mixed dyes, which meant they could be electrostatically attracted toward the negative surface of the CuS/ZnS@PVP nanofibers.^29,30^ After the photocatalytic reaction, the surface charge of the CuS/ZnS@PVP nanofibers turned back to more negative (−17.7 mV) again due to the removal of degraded products from the catalyst surface. It was also proven that no catalytic reaction took place in the dark condition. Interestingly, the reusability for the mixed-dyes degradation still remained at >95%.

**Fig. 6:**
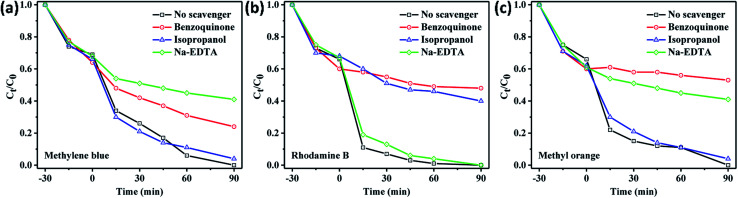
The roles of different scavengers (benzoquinone, isopropanol, and Na-EDTA) on the photocatalytic degradation of (a) methylene blue, (b) rhodamine B, and (c) methyl orange.

Furthermore, experiments involving different scavengers for active species trapping was performed to study the role of the active species in each photocatalytic dye degradation, as can be seen in Fig. 6. In this study, benzoquinone, isopropanol, and Na-EDTA scavengers were used to capture the active species of O_2_˙^−^, OH˙, and h^+^, respectively as reported elsewhere.^31–33^ When the Na-EDTA scavenger was used during the photocatalytic dye degradation of methylene blue, the final concentration still remained at around 40% after 90 min (Fig. 6a). This suggested that the photocatalytic dye degradation of methylene blue was mostly due to h^+^ in the valence band. Meanwhile, the photocatalytic degradation was highly dependent on the OH˙ and O_2_˙^−^ active species for rhodamine B, as shown in Fig. 6b. Likewise, a very slow degradation was observed when benzoquinone was used during the photocatalytic degradation of methyl orange (Fig. 6c) indicating that O_2_˙^−^ plays a significant role for the degradation.

### 3.4. Photocatalytic mechanism reaction

**Fig. 7:**
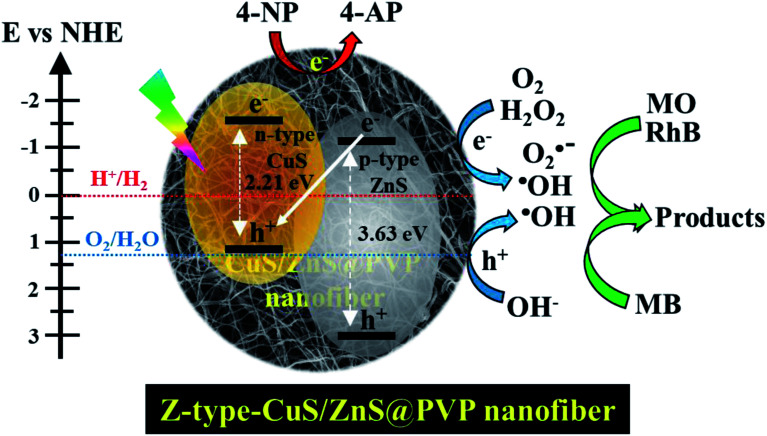
Schematic drawing of the mechanism for the conversion reaction of 4-nitrophenol and mixed dyes by CuS/ZnS@PVP nanofiber under solar light irradiation.

In this work, an inorganic photocatalyst of CuS and ZnS was simultaneously incorporated into the PVP nanofibers through the electrospinning and annealing processes as a substrate to hold the catalyst. The metal and sulfur precursors were initially in their ion forms when they were trapped in the PVP nanofibers. Afterwards, the pre-oxidation at a temperature of 180 °C was carried out to prevent the PVP from dissolving due to its natural hygroscopic properties. The following annealing process at 400 °C under the N_2_ atmosphere was to initiate the metal sulfide formation of CuS and ZnS in the nanofiber as well as to obtain the partially carbonized-PVP nanofiber. The as-prepared CuS/ZnS@PVP nanofiber revealed a better photocarrier separation due to the Z-scheme effect, which could extend the lifetime of both e^−^ and h^+^ for photocatalytic reduction and oxidation reactions. For the conversion of 4-nitrophenol, the 4-nitrophenol was first adsorbed on the nanofiber matrix, followed by the reduction once the light irradiation was introduced. Under light irradiation, the photogenerated e^−^ served as an additional source of electrons for 4-nitrophenol reduction to produce 4-aminophenol, together with efficient electron relay and separation (Fig. 7).

The reaction equation of the photocatalyst irradiated with light to generate e^−^ and h^+^ is shown in eqn (2). For the photo-oxidation reaction, holes with a high oxidative potential can either oxidize the organic dyes directly (eqn (3)), or generate the active hydroxyl radicals (HO˙) *via* water decomposition (eqn (4)), or react with hydroxyl ions (OH^−^) (eqn (5)).

2 CuS/ZnS@PVP + photon → e^−^ + h^+^

3 h^+^ + mixed-dyes → degradation products

4 h^+^ + H_2_O → H^+^ + HO˙

5 h^+^ + OH^−^ → HO˙

The radical species generated during the charge transfer are essential for photocatalytic dye degradation. Meanwhile, the photogenerated electrons for the reduction reaction in the conduction band react with adsorbed oxygen to yield superoxide anions (eqn (6)), or peroxide (eqn (7)), which subsequently form hydroxyl radicals (HO˙) (eqn (8)). The hydroxyl radical is a highly reactive and powerful species that could partially or totally decompose many organic pollutants to their degraded products (eqn (9)).^34,35^ In this work, the complete degradation of mixed dyes was indicated by obtaining a colorless solution. The complete photocatalytic reaction by CuS/ZnS@PVP nanofiber is shown in Fig. 7.

6 e^−^ + O_2_ → O_2_˙^−^

7 2e^−^ + O_2_ + 2H^+^ → H_2_O_2_

8 e^−^ + H_2_O_2_ → OH^−^ + HO˙

9 HO˙ + mixed dyes → degraded products

## 4. Conclusions

In summary, we successfully demonstrated a concept of a Z-type inorganic photocatalyst embedded in a PVP nanofiber *via* electrospinning and annealing techniques. The Z-type CuS/ZnS@PVP nanofiber is beneficial in that it absorbs both UV and visible light, hinders the recombination of photocarriers, and enhances the surface area due to the nanofiber matrix. This work applied the synthesized CuS/ZnS@PVP nanofibers for the reduction of 4-nitrophenol to 4-aminophenol and for mixed-dyes degradation under solar light irradiation with the total conversion occurring within 2 h. The photocatalyst demonstrated an easy way to ensure its reuse after the photoreaction, which also exhibited excellent stability/durability, with the percentage retention being higher than 90% after 5 cycle runs. This CuS/ZnS@PVP nanofiber is potentially applicable as a green process that could be implemented for chemical pollutants detoxification.

## Conflicts of interest

There are no conflicts to declare.

## Supplementary Material

RA-012-D2RA01476A-s001
