# Supplementary material for: Direct Z-scheme of n-type CuS/p-type ZnS@electrospun PVP nanofiber for the highly efficient catalytic reduction of 4-nitrophenol and mixed dyes
Source: RSC Adv. 2022 May 31;12(25):16165–73. doi: 10.1039/d2ra01476a (PMC9155256; doi:10.1039/d2ra01476a)
Supplement: RA-012-D2RA01476A-s001 [file RA-012-D2RA01476A-s001.pdf]

Electronic Supplementary Material (ESI) for RSC Advances.
This journal is © The Royal Society of Chemistry 2022

Direct Z-Scheme of n-type CuS/p-type ZnS@Electrospun PVP Nanofiber for Highly Efficient Catalytic Reduction of 4-Nitrophenol and Mixed-Dyes

Elvri Melliaty Sitinjak[a], Indra Masmur[b*], Vita Mey Destty Marbun[c], Poltak Evencus Hutajulu[c], Golfrid Gultom[d], and Yenny Sitanggang[a]

[a]Department of Chemical Engineering, Politeknik Teknologi Kimia Industri, Medan-20228, Indonesia

[b]Department of Chemistry, Faculty of Mathematics and Natural Sciences, Universitas Sumatera Utara, Medan-20155, Indonesia

[c]Department of Palm Oil Agribusiness, Politeknik Teknologi Kimia Industri, Medan-20228, Indonesia

[d]Department of Mechanical Engineering, Politeknik Teknologi Kimia Industri, Medan-20228, Indonesia

*Corresponding author: intar76@yahoo.com

Supplementary Information

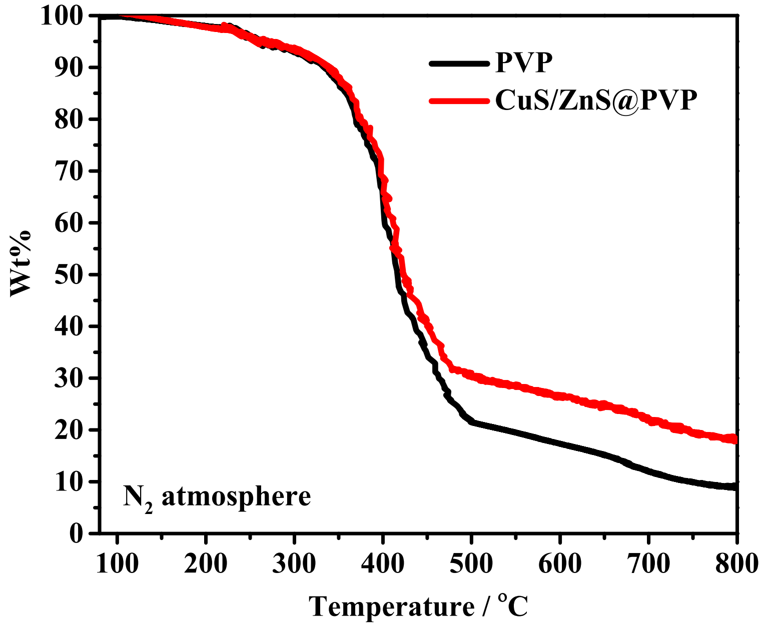

Fig. S1 TGA curves of PVP (black line) and CuS/ZnS@PVP nanofibers (red line) under N_2 atmosphere.

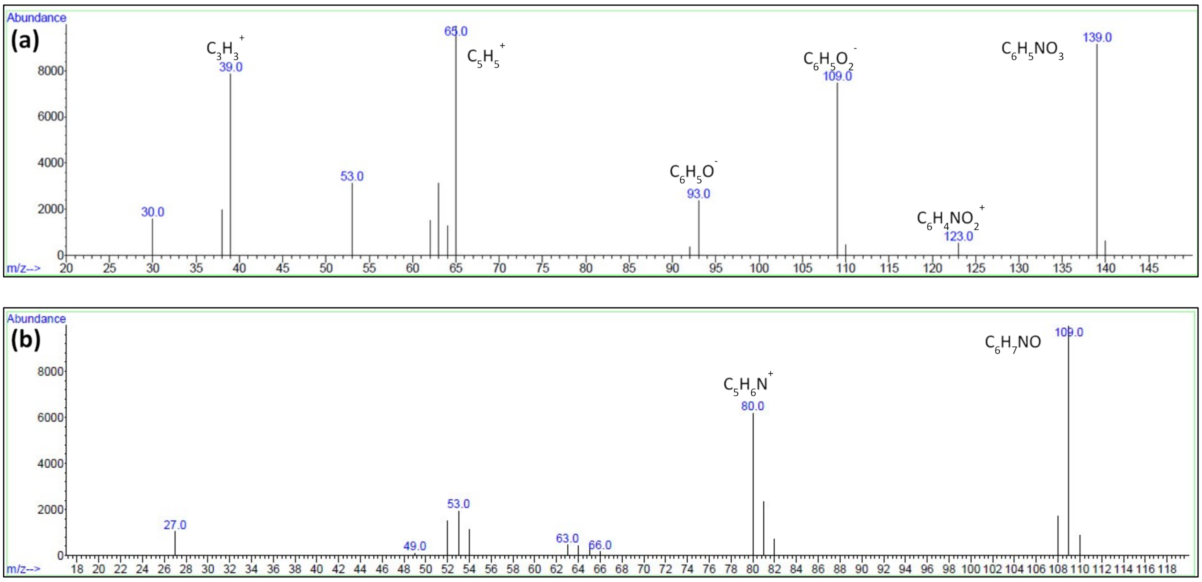

Fig. S2 The GC-MS chromatogram of (a) before and (b) after photocatalytic reduction of 4-nitrophenol for 2 h with methanol as the hole scavenger.

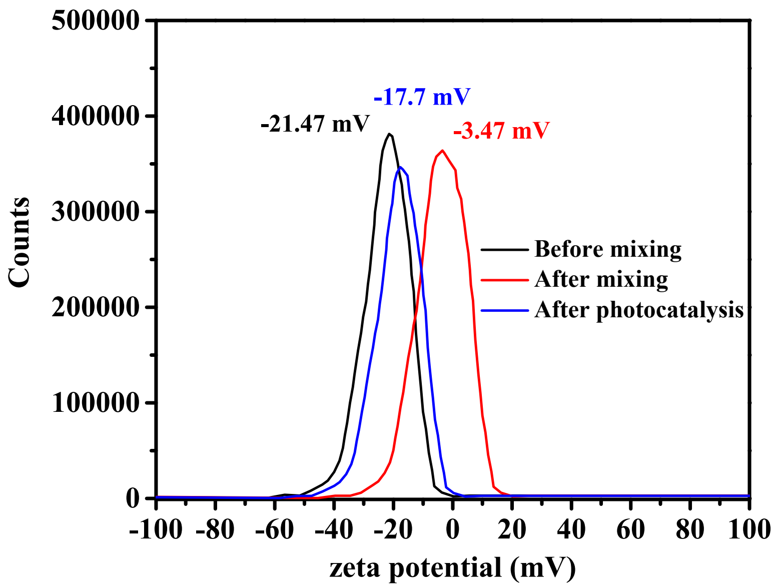

Fig. S3 Zeta potential CuS/ZnS@PVP nanofiber for sample (a) before mixing, (b) after mixing with mixed-dyes, and (c) after photocatalysis.

Table S1. The concentration of each dye during photocatalytic degradation in mixed-dyes by CuS/ZnS@PVP nanofiber

**Time (min)**	**C_t/C_0**		
	**Methylene blue**	**Rhodamine B**	**Methyl Orange**
**-30**	1	1	1
**-15**	0.73	0.73	0.73
**0**	0.67	0.67	0.67
**15**	0.51	0.11	0.21
**30**	0.33	0.07	0.14
**45**	0.19	0	0.07
**60**	0.13	0	0
**90**	0	0	0
